# Impacts of thyme and/or garlic oils on growth, immunity, antioxidant and net farm income in Damascus goats

**DOI:** 10.1038/s41598-024-62417-0

**Published:** 2024-06-07

**Authors:** Tharwat Imbabi, Tamer M. M. Hassan, Ali Osman, Ayman H. Abd El Aziz, Abuelkassem A. Tantawi, Mohammed A. F. Nasr

**Affiliations:** 1 Department of Animal Production, Faculty of Agriculture, Benha University, Benha, 13736 Egypt; 2 Biochemistry Department, Faculty of Agriculture, Zagazig University, Zagazig, 44511 Egypt; 3 Department of Animal Husbandry and Animal Wealth Development, Faculty of Veterinary Medicine, Damanhour University, Damanhour, 22511 Egypt; 4 Animal and Poultry Production Department, Faculty of Agriculture, Minia University, El-Minya, 61519 Egypt; 5 Department of Animal Wealth Development, Faculty of Veterinary Medicine, Zagazig University, Sharkia, Egypt

**Keywords:** Damascus goats, Thyme and garlic essential oil, Growth performance, Net farm income, Immunity, Antioxidant parameters, Physiology, Environmental sciences

## Abstract

This study aimed to evaluate the impact of thyme and/or garlic oil administration on growth performance, immunity, antioxidant, biochemical parameters, and net farm income of Damascus goats. Forty weaned Damascus goats were allocated into four groups. The first group was the control without oral administration, while the 2nd (Th), 3rd (Gr), and 4th (ThGr) groups were orally administrated by (2 ml/goat/day) of thyme oil, garlic oil and their mixture (1:1), respectively during the whole experiment period. The final body weight of goats orally administered oil mixture was the heaviest group, it was 10, 4.5 and 3.5% than the control, Th. and Gr. groups, respectively with better feed conversion ratio and high net farm income. Goats of ThGr. group revealed the best immunity, antioxidant and general health condition than the control group with 50% reduction of MDA. Liver (AST, 33% and ALT, 38%) and kidney (creatinine, 88%) functions improved by oils mixtures orally administration compared with the control group. LDL, triglyceride and cholesterol were reduced by 47, 33 and 21% compared with the control group, respectively. Thus, mixture oil administration (thyme and garlic at the ratio of 1:1, 2 ml/goat/day) improved growth (10%), antioxidant status (MDA 50%), liver (AST, 33% and ALT, 38%), kidney function (creatinine, 88%), the FCR (17.4%) and net farm income (21%), of Damascus goats.

## Introduction

Goats are deemed one of the enormous animals in the tropic and semi-tropic environments. They adapt well to various environmental circumstances^1^. During the last decade the world production of fresh and chilled goats' meat increased by 23% with an increase of milk (13%) and fat (25%). On the other hand, goat meat (chilled and fresh) production in Egypt decreased by 74%, while goat milk increased by 43%^2^.

Recently consumers are seeking for safe animal products without synthetic chemical residues^3,4^. Also, the European Union banned antibiotics usage in animal diets in 2006 regarding eco-friendly and healthy products^5^. Therefore, researchers and breeders are trying to find natural products for promoting ruminant growth as herbal plants and their oils^3,6–8^. Essential oils used in livestock rations^9^. They are a blend of naturally volatile and lipophilic with other substances in different values of terpene and phenylpropanoid that extracted from plants by steam and/or water distillation^10–12^.

Essential oils have an antibacterial, antifungal, antiviral, insecticidal, antioxidant, anti-inflammatory, and immune-modulatory properties that amend the rumen fermentation profiles, and consequently improve animal growth and production. This achieved by augmenting the activity of digestive enzymes, digestibility and diet absorbance^13–16^. Worldwide utilizing mixtures of different essential oils to boost animal production^17,18^. But more attention should be paid for the oil selection to form possibly effective mixtures, because they may interact with each other (additive, antagonistic or synergistic agents)^14^.

Garlic (*Allium sativum*) is a member of the family Liliaceace. It has antibacterial and antioxidant properties^19^, antihypertensive^20,21^, lipid-lowering effects^22^, enhances T-lymphocytes, natural killer cells, and interleukin-2^23^. Recently it showed a great reduction of gastrointestinal nematodes infection in goats^24^. This may be attributed to the various bioactive sulfur-containing compounds in garlic (alliin, diallyl sulfidess, and allicin) that connect with sulfhydryl groups of proteins to block the parasites’ physiological metabolism^25,26^.

Thyme is an aromatic plant that is widely allocated in Europe, Asia and North Africa. It has ethnopharmacological relevance with several bioactive components. Thyme essential oil has an effect against *Salmonella Typhimurium, Staphylococcus aureus* and Gram-positive bacteria than Gram-negative bacteria^27,28^. The economic importance of thyme is linked to its essential oils^29^. Thyme encompasses monoterpene phenols, carvacrol, thymol and p-cymene, α-pinene, 1,8-cineole, camphor, linalool, borneol and γ-terpinene^27,30^. Thymol (5-methyl-2-isopropyl phenol) is one of the main components of thyme and oregano essential oils^27,31^. To our knowledge, there were few studies on this issue on goats. Therefore, the current study aimed to evaluate the potential impact of thyme and garlic oil administration on Damascus goats' growth performance, immunity, antioxidant, biochemical parameters, and net farm income.

## Materials and methods

All experimental procedures were accomplished with reference to the local Experimental Animal Care Committee and approved (20209) by the Institutional Committee of the Department of Animal Production, Faculty of Agriculture, Benha University, Egypt.

### Diet, experimental design and management

This study was carried out for three months (February to April 2021) at the experimental farm of the Faculty of Agriculture, Benha University, Egypt with 40 weaned female Shami goats (Damascus) with 17.86 ± 0.22 kg average body weight and 6–8 months of age. Goats were fed on a basal diet and classified to four groups (10 goats in each group), the first group was the control (cont; without any oral administration); the second (Th), third (Gr) and fourth group (ThGr) were orally administrated by thyme oil (2 ml/goat/day), garlic oil (2 ml/goat/day) and mixed oil (1 ml thyme + 1 ml garlic/goat/day), respectively just before feeding during the whole experiment period (3 months). Goats were housed in individual pens (0.97 × 2.82 m) bedded with straw.

All experimental goats were individually weighed at the beginning of the experiment and every month until the end of the experiment to the nearest 10 g. The average daily gain (ADG) was calculated using this formula: ADG = Final body weight (kg) − Initial body weight (kg)/experimental duration (days). Both consumed diets and refusals (if any) were recorded daily. Total and daily feed intake (TFI, DFI) and feed conversion ratio (FCR) were then calculated. Feed conversion ratio (FCR) = Total feed intake (kg)/total weight gain (kg). Goats were housed individually in hygienic metallic pens and supplied with clean water all time. The routine vaccination program against infectious diseases and medications against internal and external parasites were applied.

The basal diet was formulated according to the recommendations of NRC^32^ (Table 1). The main components of the thyme and garlic essential oil were represented in Tables 2 and 3, respectively). A Hewlett-Packard II 5890 gas chromatography (GC) system with an FID detector and an HP-5 ms capillary column (30 m × 0.25 mm, film thickness 0.25 m) was used to analyze the essential oils. The temperatures of the injector and the detector were set to 290 °C and 220 °C, respectively. The GC stove temperature was raised from 60 to 240 °C at 3 °C min^−1^ and held isothermally for 10 min. At a flowrate of 1 ml min^−1^, helium served as the carrier gas. Samples diluted to 1:100 in diethyl ether by volume; in the spitless mode were manually injected. Quantitative information was acquired electronically from FID region rate information without the utilization of amendment factors. Individual component relative percentages expressed as percent peak area compared to the total composition of the EO as determined by GC–MS analysis^33,34^.

**Table 1:** Goats’ diet with its chemical composition.

Ingredients (g/kg)	Goat’s diet
Yellow corn	310
Wheat bran	50
Wheat straw	230
Soybean meal	180
Cotton seed meal	40
Molasses	50
Egyptian clover	100
Sodium chloride	10
Calcium carbonate	5
Limestone	10
Di-calcium phosphate	5
Ammonium chloride	5
Vitamins and minerals mixture^a^	5
Chemical composition (g/kg)	
Dry matter	904.5
Crude protein	151.6
Ether extract	35.0
Nitrogen-free extract (NFE)^b^	545.0
Crude fiber	159.6
Neutral detergent fiber (NDF)	323.5
Acid detergent fiber (ADF)	206.7
Ash	108.8
Metabolizable energy (ME, Mcal/kg DM)^c^	2.71

^a^Each 1 kg of vitamins and minerals mixture contains: Vit. A = 4,000,000 IU, Vit. D_3_ = 833,333.33 IU, Vit. E = 5000 mg, Zn = 20,000 mg, Mg = 23,333.33 mg, Fe = 20,000 mg, Cu = 10,000 mg, I = 1666.66 mg, Se = 100 mg, Co = 333.33 mg and Calcium carbonate up to 1 kg.

^b^NFE calculated as [100-(CP + EE + ash + CF)].

^c^ME (MJ/kg DM) = 0.012 CP + 0.031 EE + 0.005 CF + 0.014 NFE (calculated according to MAFF, 1975) and changed to Mcal (* 4.184).

**Table 2:** The main components of the thyme essential oil.

Component	Concentration (%)
2-Octen-1-ol	0.68
β-Myrcene	1.41
p-Cymol	0.17
Eucalyptol	1.33
γ-Terpinene	7.54
cis-Sabinene hydrate	0.72
Borneol	3.42
1-Terpinen-4-ol	0.86
α-Terpineol	1.06
Thymol methyl ether	0.81
Thymol	38.68
Carvacrol methyl ether	1.77
Carvacrol	6.4
β-Caryophyllene	3.77
δ-Cadinene	1.53
Caryophyllene oxide	1.56
Linalool	3.3

Gas chromatography/mass spectrometry (GC–MS) was used to examine the chemical components of thyme essential oil.

**Table 3:** The main components of the garlic essential oil.

Component	Percentage (%)
Diallyl sulfide	4.93
Methyl allyl disulfide	5.96
Dimethyl trisulfide	3.10
Allyl (Z)-1-propenyl disulfide	3.70
Methyl allyl trisulfide	17.13
3-Vinyl-[4H]-1,2-dithiin	0.85
Vinyl-[4H]-1,3-dithiin	3.20
Diallyl trisulfide	27.13
Diallyl tetrasulfide	2.60
Diallyl disulfide	25.50

Gas chromatography/mass spectrometry (GC–MS) was used to examine the chemical components of garlic essential oil.

### Chemical analysis

Goat’s diet was analyzed according to AOAC^35^. Dry matter (DM) was measured by a hot air circulation oven (Heraeus Ut20, Germany) at 105 °C for four hours (method no. 930.15). Ash was measured with a Burnout furnace Ney Vulcan D-550, USA (method no. 942.05). Crude protein (CP) was measured with Kjeltec^®^ system 2100, FOSS-Sweden (method no. 984.13), and ether extract (EE) using Soxtec^®^system 2045, FOSS-Sweden (method no. 920.39). Neutral detergent fiber (NDF) and acid detergent fiber (ADF) were measured^36^. Organic matter (OM) was measured from the difference between DM and ash.

### Net farm income

Finance analysis has been done to assess and compare the profitability among the various groups. The net farm income (NFI) was assessed^37^ from the difference between total income (TI) and total costs (TC). TI and TC were individually assessed for each goat. TC include the feeding costs (diet plus oral oils), the labor costs (the price farmers’ working hour multiplied by the number of hours employed per goat^38^, veterinary care costs (vaccines, and veterinary supervision). Water, electricity, and equipment maintenance in addition to costs of litter and building rent value were also calculated per goat for each group. TI consisted of income value from marketable goats’ body weight gain. All these variables were calculated in Dollars.

### Blood sampling and analysis

Blood samples were collected at the end of the experiment from all experimental goats in the morning just preceding feeding from the jugular vein to a clean dry test tube. The samples were left at room temperature for 45–65 min and then centrifuged for 30 min at 4000 rpm. Serum was separated into clean dried glass vials (5–7 ml) and stored frozen (− 20 °C) until analysis. Blood was analyzed for total protein^39^, albumin^40^, globulin (by subtracting the albumin value from the total protein concentration), urea^41^, creatinine, ALT and AST^42^ with commercial kits (Wako Pure Chemical Industries, Osaka, Japan). Single radial immune diffusion technique was used to quantify total immunoglobulin IgA, IgM, IgG in blood serum (bind ARIDtm Blinding site limited, Birmingham, UK) according to the method described by Fahey and McKelvey^43^. Serum malondialdehyde (MDA) level was determined^44^. Serum Superoxide Dismutase (SOD) activity was assayed at 420 nm on a UV–Vis Shimadzu spectrophotometer (2450)^45^. Catalase activity (CAT) was measured by the spectrophotometric method based on the decomposition of H_2_O_2_^46^. Blood LDL, HDL, triglyceride, and cholesterol were measured^47^ with commercial kits (Wako Pure Chemical Industries, Osaka, Japan).

### Statistical analysis

The data were analyzed using one-way analysis of variance (ANOVA) (SAS: Statistical Analysis System software version 2004). Shapiro–Wilk tests has been used to test the data normality and it was normally distributed. Duncan’s multiple-range tests was used to determine the significance differences among groups. The applied static model is as follows:

$$ {\text{yij }} = \, \mu \, + {\text{ Ti }} + {\text{ eij}} $$

Where yij is the observations, µ = general mean, Ti is the effect of i_th_ diet supplementation, where i = 1, 2, 3, and 4 and eij is the random error.

### Ethical approval

All experimental procedures were accomplished with reference to the local Experimental Animal Care Committee and approved (20209) by the Institutional Committee of the Department of Animal Production, Faculty of Agriculture, Benha University, Egypt. The current study is reported in accordance with ARRIVE guidelines.

## Results

The thyme and garlic oil orally administration improved the growth performance, weight gain, average daily gain, feed conversion ratio, and net farm income. The final body weight of goats orally supplemented with oil mixture of thyme and garlic was the heaviest group. It was 10, 4.5 and 3.5% than the cont, Th. and Gr. groups, respectively. The final weight gain and the average daily gain were the best of the goats orally supplemented with mixture of garlic and thyme oil. They were 12.55 kg and 0.14 kg/day, respectively. Moreover, their feed conversion was better (17, 11 and 7%) with high net farm income (21, 11.5 and 7%) compared with the cont, Th. and Gr. groups, respectively (Table 4).

**Table 4:** Effect of oral administration of thyme and/or garlic on growth performance, feed intake, feed conversion and economic feed efficiency of weaned Damascus goats.

Parameter	Control	Thyme	Garlic	Thyme and garlic	SEM	P-value
Body weight (BW) (kg)						
IBW	17.78	18.00	17.92	17.75	0.86	0.99
BW1	20.97	21.80	22.03	21.88	0.83	0.58
BW2	24.32^b^	25.78^ab^	25.98^ab^	26.38^a^	0.72	0.04
FW	27.57^b^	28.98^ab^	29.26^ab^	30.30^a^	0.66	0.004
Weight gain (WG) (kg)						
WG1	3.19^b^	3.80^a^	4.11^a^	4.13^a^	0.16	< 0.001
WG2	3.35^b^	3.98^ab^	3.95^ab^	4.50^a^	0.23	< 0.001
WG3	3.25	3.20	3.28	3.92	0.37	0.21
FWG	9.79^c^	10.98^b^	11.34^b^	12.55^a^	0.39	< 0.001
Average daily gain (ADG) (kg/day)						
ADG1	0.113^b^	0.135^a^	0.146^a^	0.147^a^	0.02	< 0.001
ADG2	0.108^b^	0.128^ab^	0.127^ab^	0.145^a^	0.03	0.001
ADG3	0.108	0.106	0.109	0.130	0.05	0.20
ADG	0.110^c^	0.123^b^	0.127^b^	0.141^a^	0.01	< 0.001
Total feed intake (TFI) (kg)						
TFI1	17.42	17.75	17.80	18.15	0.36	0.56
TFI2	22.40	23.81	23.60	24.25	0.36	0.56
TFI3	25.99	26.56	26.19	27.28	0.36	0.56
TFI	65.81	68.12	67.59	69.68	1.07	0.56
Daily feed intake (DFI) (kg/day)						
DFI	0.73	0.76	0.75	0.78	0.04	0.55
Feed conversion ratio (FCR) (feed/gain)						
FCR1	5.46^a^	4.67^b^	4.33^b^	4.39^b^	0.35	< 0.001
FCR2	6.68^a^	5.98^ab^	5.97^ab^	5.38^b^	0.46	0.003
FCR3	7.99	8.30	7.98	6.95	1.76	0.76
FCR	6.72^a^	6.20^ab^	5.96^b^	5.55^b^	0.39	0.002
NFI	2.53^b^	2.74^ab^	2.85^ab^	3.06^a^	0.24	0.003

*SEM* Standard error mean, *BW* Body weight, *WG* Weight gain, *ADG* Average daily gain, *TFI* Total feed intake, *FCR* Feed conversion ratio.

*NFI* Net farm income during the 3 months of the experimental period (Benefit/cost ratio) with 5.66 and 0.33$ for each kg of Shami goats body weight and feed intake, respectively.

^a,b,c^Means within the same row with different superscript letters differ significantly (p ≤ 0.05).

Thyme and garlic oils improved the goats’ immune status with better antioxidant levels. Goats of the ThGr group revealed the best immunity, antioxidant and general health condition. IgA (21, 7 and 7%), SOD (62, 51 and 43%), CAT (27, 24 and 12%) and TAC (30, 25 and 21%) were better than the cont, Th and Gr groups, respectively with a reduction of MDA by 50, 37 and 18%, respectively. The liver and kidney functions improved by thyme and garlic oils orally supplementation. AST (33, 22 and 24%), ALT (38, 23 and 18%) and creatinine (88, 18 and 34%) levels were reduced compared with the other three groups, respectively (Table 5). The total protein and globulin were the highest in the group orally supplemented with the oil mixture compared with the other experimental groups with better HDL in Gr. and ThGr. groups. While LDL was the lowest in ThGr. group. Concurrently, the triglyceride and cholesterol were reduced by 33 and 21% compared with the cont group, respectively (Table 5).

**Table 5:** Effect of oral administration of thyme and/or garlic on serum immunity, antioxidant and biochemical parameters of weaned Damascus goats.

Parameters	Control	Thyme	Garlic	Thyme and garlic	SEM	P-value
IgA (mg/mL)	36.00^c^	40.50^b^	40.50^b^	43.50^a^	0.25	< 0.001
IgM (mg/mL)	8.50	8.00	8.00	9.00	0.33	0.16
IgG (mg/mL)	158.50	172.00	171.50	171.50	11.97	0.82
SOD (Ul/L)	0.96^d^	1.25^c^	1.45^b^	2.55^a^	0.03	< 0.001
MDA (U/L)	1.77^a^	1.42^b^	1.09^c^	0.89^c^	0.21	< 0.001
CAT (U/L)	2.81^c^	2.88^c^	3.17^b^	3.56^a^	0.03	< 0.001
TAC (mmol/L)	10.39^b^	10.70^b^	11.05^b^	13.35^a^	0.22	< 0.001
AST (U/I)	31.50^a^	27.00^a^	26.00^a,b^	21.00^b^	1.23	0.002
ALT (U/I)	29.00^a^	23.50^b^	22.00^c,b^	18.00^c^	1.01	< 0.001
Creatinine (mg/dl)	7.06^a^	1.05^c^	1.31^b^	0.86^d^	0.77	< 0.001
Urea (mg/dL)	32.50	30.50	41.50	36.00	3.41	0.19
Total Protein (g/dL)	7.15^b^	7.30^b^	7.45^b^	8.15^a^	0.13	0.002
Albumin (g/dL)	3.75^a,b^	3.40^b^	4.10^a^	3.50^b^	0.08	< 0.001
Globulin (g/dL)	3.40^c,b^	3.90^b^	3.35^c^	4.65^a^	0.12	< 0.001
LDL-c (mg/dL)	21.70^a^	21.50^a^	14.70^b^	11.60^c^	0.61	< 0.001
HDL-c (mg/dL)	42.50^b^	44.00^b^	50.00^a^	51.50^a^	0.74	< 0.001
Triglyceride(mg/dL)	15.00^a^	9.50^b^	9.00^b^	10.00^b^	0.72	< 0.001
Cholesterol (mg/dL)	75.00^a^	57.50^b^	75.00^a^	59.00^b^	0.43	< 0.001

*SEM* Standard error mean, *MDA* Malondialdehyde, *CAT* catalase, *SOD* superoxide dismutase, *TAC* total antioxidant capacity, *IgG* Immunoglobulin G, *IgA* Immunoglobulin A, *IgM* Immunoglobulin M, *AST* Aspartate aminotransferase, *ALT* Alanine aminotransferase, *LDL-c* low-density lipoprotein-cholesterol, *HDL-c* High-density lipoprotein-cholesterol.

^a,b,c^Means within the same row with different superscript letters differ significantly (p ≤ 0.05).

## Discussion

Thyme, garlic and their essential oils are natural alternatives that may be used for growth promoters and antibiotics in ruminant rations^6–8^. Essential oils can be used in dairy cows^9^. This research aimed to evaluate the potential impact of thyme and garlic oil orally administration to Damascus goats on growth performance, immunity, antioxidant, biochemical parameters and net farm income.

It is tremendously challenging to compare the investigations that used various essential oils since the outcomes will be established on several issues, such as oil composition, doses, extraction methods, route of administration, animal age and housing. The mixture of thyme and garlic oils improved the growth performance of Damascus goats. It improved final body weight, ADG and FCR. These results were supported by others on rabbits and lambs^48,49^. Zhong et al.^49^ reported that garlic dietary supplementation augmented the average daily gain in lambs. Moreover, Abdel-Wareth and Metwally^48^ stated that the daily weight gain and FCR of male Californian rabbits were improved by thyme supplementation without any effect on daily feed intake. The current findings did not show any significant variations among the various groups regarding total and daily feed intake supported by others in beef cattle^50,51^. There were conflicting results regarding FCR. Some authors reported that thyme and/or garlic did not affect FCR^49,52–54^, while others detect an improvement^48^. The current findings were comparable with Abdel-Wareth and Metwally^48^. The reported feed intake, ADG and FCR of the current study were similar to that performed on goats^53^.

The gastrointestinal nematodes infestation of small animals reduced the average daily gain (23–63%) in lambs^55^ and feed intake^56^, feed digestion in calves^57^, meat quality in lambs^58^ with an increase of FCR in sheep^59^. Therefore, thyme and/or garlic supplementation reduced the parasitic load and consequently improved the growth and productive performance of treated calves than the control group^57^. Moreover, there was an increase in the goats’ live weight treated with anthelmintic than in the control group. Dietary garlic oil supplementation did not affect feed intake and average daily gain of lambs without parasitic infestation^60^. While Hasan et al.^24^ reported an increase in average daily gain (10.3%) of grazing goats suffered from internal parasites if they were supplemented orally with garlic solution^24^.

The current study revealed that the oil mixture increased the final body weight by 10% which was comparable to Hasan et al.^24^ on grazing goats. This improvement may be attributable to several factors (a) the anthelmintic effect of the thyme and garlic oils that reduced the gastrointestinal parasites which hampering the growth of small ruminants leading to economic losses^61,62^, (b) the power antimicrobial activity against gram-positive and negative bacteria through disruption the bacterial plasma membrane and glucose uptake reduction, while the beneficial bacteria are less sensitive to the inhibitory effects of garlic^27,63,64^, (c) thyme methanolic extract may influence the fermentation efficiency of rumen and may be used as a mitigating mediator of methane^65^, (d) improved the digestibility and feed efficiency of growing lambs^66^ through activation of ruminal bacteria, stimulation the secretion and flow of bile, saliva and gastric enzymes^67–69^ that may modify the microbial numbers in the rumen and decreased the of dietary protein degradation with increasing N escape and protein flow to the small intestine, consequently improved the nitrogen utilization^70,71^, (e) increased the synthesis and activity of pancreatic enzymes, enriching digestion, absorption and utilization of proteins in the small intestine^72^, (f) reducing the proportion of acetate with increasing the propionate^73^, (g) thymol may protect the microvilli^74^. The current study revealed that ThGr groups was the best regarding the net farm income. Better performance of the animal such as weight gain and FCR will providing higher incomes, total profit margin with reducing production costs^75,76^.

El-Azrak et al.^77^ reported an improvement in the immune response and serum IgG following essential oil supplementation of goats which supported the current results^78^. This may be owned to the increasing phagocytosis as observed in pigs^79^. Moreover, the essential oils may be reduced the animal stress because the stress reduced the release of immunoglobulins^80^. Releasing somatostatin and adrenal corticosteroid hormones are accountable for lowering the immunoglobulins production^80^. Blood parameters are imperative tool for detecting the metabolic syndromes^81^ and to estimate the physiological state of animals^82^. MDA and glutathione are the foremost marker of lipid peroxidation utilized for assessing the oxidative damage^83,84^. Antioxidant enzymes (SOD) accomplish an imperative role of antioxidant defenses^85^. Stress and high lipid peroxidation increased the production of ROS that increased oxidative destruction and MDA level with a reduction of the antioxidant enzymes activity^86–88^. Thymol boosts the total antioxidant condition in vivo^89^ due to it improves the activity of antioxidant enzymes, such as superoxide dismutase and catalase^90^.

Liver is an imperative organ in metabolic body processes. Serum Transaminases activities were represented the damage and recovery of the liver cells and the pathological condition^91,92^ that are subtle to the toxic matters^93^. This increase can be ascribed to liver or muscle damage, septicemia, and/or toxemia and their values are associated with the severity of cell damage^94^. Aba et al.^95^ stated that the normal level of goats AST (IU/l), ALT (IU/l), albumin (g/dl) and cholesterol (mg/dl) were 28.60–92, 2.59–29.65, 2.75–3.86 and 33.84–132.42, respectively. The oral administration of phytobiotics enhanced the liver function of rabbits^96^ which supported the current findings. Ghoneem and Mahmoud^97^ revealed that there was an increase of the AST (2.5%) and ALT (10%) levels with thyme essential oil supplementation in Damascus goats. But Abdel-Wareth and Metwally^48^ detected a reduction of AST and ALT in male rabbits. Moreover, Usur^53^ stated that essential oil reduced AST and ALT by 8 and 11%, respectively in goats. Vakili et al.^98^ reported that essential oils supplementation did not influence on hepatic enzymes^67,99,100^.

The creatinine level of the current study was comparable to that reported by Ghoneem and Mahmoud^97^. Thyme and/or garlic supplementation increased the serum creatinine and urea in goats^53,67,97^. On the other hand, this study revealed a reduction of creatinine level that supported by Abdel-Wareth and Metwally^48^ in male rabbits. But others did not detect any difference^98,101–103^ in ewes, lambs and calves. Essential oils did not influence the serum urea level that was supported by other^77^ in goats. The creatinine levels in this investigation are within the normal reported range (0.6–1.1 mg/dl)^1,104^.

The current finding regarding the levels of total protein, albumin and globulin was similar to the findings of others in goats^1,53,97^. There have been conflicting results on the effect of orally supplemented mixture of thyme and garlic oil on the total protein, albumin and globulin. Several authors have detected an increase of their levels^105–107^. While others have stated that there were no differences following thyme and/or garlic supplementation^77,97,108,109^. The current outcomes were similar with the findings of the research that stated an increase of total protein and globulin after orally administration of thyme and/or garlic oil.

The high levels of total protein and globulin in goats treated with oil mixture may be attributable to; (a) the serum protein was positively associated with body weight and protein manufacture^110,111^ and food protein^101^, (b) they stimulate liver cells to multiplication the protein synthesis^109^, (c) the improvements of ruminal microbial protein synthesis^73^ and crude protein digestibility^97^. On the other hand, there was a reduction of total protein followed ginger oil supplementation due to the modification effect of essential oils on the ruminal microbial population status and decreasing the contribution of *Prevotella* spp. that is chiefly accountable for protein degradation and amino acids deamination and consequently, protein metabolism^112^.

There have been inconsistent outcomes on the influence of orally supplemented thyme and/or garlic on goats' cholesterol and triglycerides. Whereas several authors have reported that this supplementation reduced cholesterol and triglycerides^53,97,113,114^ in goats. Others did not detect any difference^67,98,101,108,115^ in calves and lambs. While others reported an increase of cholesterol and triglycerides^77^ in goats. Nonetheless, these results agreed with the majority of studies that have reported a reduction cholesterol and triglycerides.

Cholesterol was responsible for the level of triglycerides in the blood. Thyme and garlic supplementation reduced cholesterol and triglycerides by 11 and 18%^97^ in Damascus goats, 11 and 7%, respectively^53^ in goats. While the current study revealed 21 and 33% reductions of cholesterol and triglycerides, respectively. These results may be attributed to; (a) decreasing the efficiency of enzymes or preventing the thiol enzymes such as HMGCoA^24^ and CoASH^116^ in liver, (b) the hypolipidemic and hypercholesterolemia characters of aromatic plants and their essential oil^117^, (c) the metabolism of herbal plants product might prevent the production of 3-hydroxy-3-methylglutaryl CO. A enzyme, which influences the cholesterol synthesis^118^. Moreover, the HDL was increased by 21% followed the orally administration of the oil mixture that may be owned to decrease the values of triglycerides, cholesterol and LDL^119^. Our finding regarding HDL was comparable with Meteab et al.^1^ who reported 39.06 mg/dl of HDL in Damascus goat.

## Conclusions

Recently consumers are seeking for safe animal products without synthetic chemical residues. Therefore, researchers and breeders are trying to find natural alternatives for growth promoters and antibiotics in ruminant rations as herbal plants and their essential oils. Thus, mixture oil administration (thyme and garlic at the ratio of 1:1, 2 ml/goat/day) improved growth (10%), antioxidant status (MDA 50%), liver (AST, 33% and ALT, 38%), kidney function (creatinine, 88%), the FCR (17.4%) and net farm income (21%), of Damascus goats. Therefore, this study recommended supplementing goats with the mixture of thyme and garlic oil for better growth and health.

## Data Availability

The data is available on request. Dr.Tharwat Imbabi Tharwat.mohamed@fagr.bu.edu.eg.
